# Daily interruption of sedation in critically ill children: study protocol for a randomized controlled trial

**DOI:** 10.1186/1745-6215-15-55

**Published:** 2014-02-13

**Authors:** Nienke J Vet, Saskia N de Wildt, Carin WM Verlaat, Catherijne AJ Knibbe, Miriam G Mooij, Wim CJ Hop, Joost van Rosmalen, Dick Tibboel, Matthijs de Hoog

**Affiliations:** 1 Intensive Care, Erasmus MC - Sophia Children’s Hospital, Dr. Molewaterplein 60, 3015 GJ Rotterdam, The Netherlands; 2 Department of Pediatrics, Erasmus MC - Sophia Children’s Hospital, Rotterdam, The Netherlands; 3 Department of Pediatric Surgery, Erasmus MC - Sophia Children’s Hospital, Rotterdam, The Netherlands; 4 Intensive Care, Radboud University Nijmegen Medical Center, Nijmegen, The Netherlands; 5 Department of Pharmacology, Leiden/Amsterdam Center for Drug Research, Leiden University, Leiden, The Netherlands; 6 Clinical Pharmacy, St Antonius Hospital, Nieuwegein, The Netherlands; 7 Department of Biostatistics, Erasmus MC, Rotterdam, The Netherlands

**Keywords:** Pediatrics, Critical illness, Sedation, Daily sedation interruption

## Abstract

**Background:**

In adult patients who are critically ill and mechanically ventilated, daily interruption of sedation (DSI) is an effective method of improving sedation management, resulting in a decrease of the duration of mechanical ventilation, the length of stay in the intensive care unit (ICU) and the length of stay in the hospital. It is a safe and effective approach and is common practice in adult ICUs. For critically ill children it is unknown if DSI is effective and feasible. The aim of this multicenter randomized controlled trial is to evaluate the safety and efficacy of daily sedation interruption in critically ill children.

**Methods/Design:**

Children between 0 and 18 years of age who require mechanical ventilation, with an expected duration of at least 48 h and need for sedative infusion, will be included. After enrollment patients will be randomly assigned to DSI in combination with protocolized sedation (intervention group) or protocolized continuous sedation (control group). A sedation protocol that contains an algorithm for increasing and weaning of sedatives and analgesics will be used. The sedative infusion will be restarted if the patient becomes uncomfortable or agitated according to the sedation protocol. The primary endpoint is the number of ventilator-free days at 28 days.

**Trial registration:**

NTR2030

## Background

Critically ill children are often sedated in order to prevent discomfort or anxiety and to facilitate care. The sedative drug of choice for the majority of critically ill children is midazolam, often given together with analgesics such as morphine or fentanyl [1]. Doses are individually titrated, based on sedation assessments, to reach the optimal level of sedation. Both inadequate and excessive sedation may have deleterious effects. Oversedation delays recovery, promotes tolerance and leads to distressing symptoms on withdrawal of the drugs [2]. Undersedation may result in increased distress and increased adverse events, such as unplanned extubation, accidental displacement of catheters and fighting the ventilator.

Despite the use of sedation algorithms, excessive sedation is a common problem in critically ill children receiving continuous sedation [3]. In adults, the administration of sedatives by continuous infusion is an independent predictor of a longer duration of mechanical ventilation as well as a longer stay in the intensive care unit (ICU) and in the hospital overall [4].

In adults, daily sedation interruption (DSI) improves clinical outcome. Every day, sedative drug infusions are interrupted and patients are allowed to ‘wake up’ from their medicine-induced sleep. During this period, patients are assessed for neurological recovery and readiness for extubation, or resedated if required [5]. In adult intensive care patients, DSI resulted in a significant decrease in the duration of mechanical ventilation, the length of stay in the ICU and the length of stay in the hospital [6]. DSI is also a safe approach: self-extubation and removal of catheters did not occur more frequently in patients treated with DSI. Follow-up studies showed that DSI reduces the incidence of complications associated with mechanical ventilation and reduced symptoms of post-traumatic-stress disorder (PTSD) [7,8]. In the last few years, some studies have confirmed the safety and efficacy of DSI [9,10], while other studies did not find a positive effect of DSI on clinical outcome [11,12]. Nevertheless, DSI is now routine practice in adult ICUs [5,13]. An even more drastic approach of no sedative drugs at all has also been shown to improve clinical outcome in adult intensive care patients [14].

For critically ill children, it is unknown if DSI is effective, feasible and safe. Data from adult ICU studies cannot be automatically extrapolated to children. Important differences in the use of sedative drugs between children and adults have been described. In adult ICU patients, propofol and remifentanyl are the drugs of choice, besides midazolam, morphine or fentanyl. In children, propofol is contraindicated for prolonged (>24 h) sedation because of the risk for propofol infusion syndrome [1]. Another important difference is that the elimination half-life of many drugs varies between adults and children, due to age-related changes in drug metabolism and renal excretion. Also, the assessment of the sedative level differs between adults and children. For example, to assess wakefulness adult patients are asked to perform actions on request, such as squeeze a hand or stick out their tongue. In most pediatric ICUs (PICUs), 80% of admissions are children <3 years of age. Younger children cannot perform such instructions on request and the assessment of their sedation level should include other parameters, such as non-verbal communication. Specific instruments, such as the COMFORT scale, have been developed and validated for assessing sedation levels in critically ill children [15]. Finally, since younger children cannot clearly communicate, their behavior is different and there might be a greater intolerance of discomfort.

We identified two studies evaluating the feasibility of DSI in children. In a pilot study in 30 ventilated children DSI was compared with standard care [16]. DSI appeared feasible and safe (similar rate of unintended extubations and line removals) and reduced the amount of sedatives administered. However, this trial was not sufficiently powered to detect differences in clinical outcomes. The second study performed by our group showed that in 20 neonates on extracorporeal membrane oxygenation (ECMO), midazolam and morphine could be discontinued following cannulation for a median of 10 h without adverse events [17].

Recently, a study was published comparing DSI with continuous sedation in children on mechanical ventilation [18]. This study showed that DSI also improves outcomes in pediatric patients. The length of mechanical ventilation and duration of intensive care stay were significantly reduced in the interrupted sedation group (10.3 vs 7.1 days, *P* = 0.021 and 14.1 vs 10.7 days, *P* = 0.002, respectively). There were no differences in adverse events between groups. Given the large differences in patient population and ICU practices between this Indian ICU and the Western setting, these results need further validation [19]. In this Dutch multicenter study, efficacy and safety of daily interruption of sedation in critically ill children will be investigated.

## Methods/Design

In this multicenter randomized controlled trial, we will compare DSI combined with protocolized sedation with standard of care (protocolized sedation only). This study is a collaborative study between PICUs in The Netherlands.

### Study population

Patients will be recruited from five tertiary medical-surgical PICUs (Erasmus MC - Sophia Children’s Hospital, Radboud University Nijmegen Medical Centre, Academic Medical Centre of Amsterdam, Leiden University Medical Centre and University Medical Centre Groningen).

Children between 0 and 18 years of age admitted to the pediatric intensive care unit, who require mechanical ventilation, with an expected duration of at least 48 h, and need for sedative drugs can be included.

Inclusion criteria: age between 0 to 18 years, at least 37 weeks of post conceptual age, anticipated duration of mechanical ventilation of at least 48 h, need for sedative/analgesic drugs.

Exclusion criteria: anticipated death within 48 h or withdrawal of life support, patients in whom level of sedation cannot be scored due to underlying neurologic condition, neurological, respiratory or cardiac instability that may not tolerate inadequate sedation (for example, traumatic brain injury, pulmonary hypertension), therapeutic hypothermia after cardiopulmonary resuscitation, difficult airway, fixed duration of mechanical ventilation, admission for ECMO, admission to our PICU after transfer from another PICU where the patient is already ventilated/sedated for >2 days, withdrawal of informed consent.

### Randomization

Within 24 h after intubation, parental informed consent will be obtained. The morning after enrolment, patients will be randomly assigned in a 1:1 ratio to DSI combined with protocolized sedation (intervention group) or protocolized sedation alone (control group).

Stratified randomization will be used in combination with random permuted blocks. Randomization will be stratified with regards to age in three groups: 0 up to 30 days, 30 days up to 2 years, and 2 years up to 18 years. A biostatistician will carry out computer randomization in advance. During the study period, the pharmacist will have access to group allocation for preparation of study medication, and each assignment is designated on a paper enclosed in a numbered, opaque sealed envelope. After informed consent is obtained, the appropriate envelope is placed in a study binder at the patient’s bedside.

### Intervention

After enrollment, patients will be randomly assigned to one of two strategies: protocolized continuous sedation combined with daily interruption of infusion of sedatives beginning 24 h after start of infusion (intervention group) or protocolized continuous sedation alone (control group).

#### Protocolized sedation/standard of care

All study centers use a standardized sedation protocol that contains an algorithm for increasing and weaning of sedatives and analgesics. It standardizes sedation management and allows nurses to adapt medication based on validated sedation scores (COMFORT behavior scale (COMFORT-b), Nurse Interpretation of Sedation Score (NISS)). The COMFORT-b is an adapted version of a scale that was originally developed by Ambuel and colleagues in 1992 for the assessment of distress in pediatric patients, except for premature neonates and children with neurological diseases and limited motor function [20]. It consists of six behavioral items: alertness, agitation, crying or in case of artificial ventilation breathing reaction, body movements, muscle tone and muscle tone in the face. A trained intensive care nurse observes a patient for a 2-minute period, during which all items are assessed on a five-point numerical scale (scored 1 to 5). The most distressed behavior during the 2-minute period is scored. The total COMFORT-b score is the total of all item scores, with a minimal score of 6 and a maximal score of 30. The cutoff points for sedation scores were established [21]. In all participating PICUs, nurses have been trained to use this scale. Interobserver variability was satisfactory, with Cohen’s κ >0.65 for all nurses.

Upon admission to the ICU patients are evaluated for the need of sedatives and analgesics according to standard medical treatment protocols. In this protocol, initially, midazolam is titrated (up to 300 μg/kg/h) according to predefined COMFORT-b scores. Adequate sedation is defined as a COMFORT-b score ≥11 and ≤22. A COMFORT-b score of <11 implies oversedation, a score >22 undersedation. When sedation is considered insufficient, morphine (up to 30 μg/kg/h) is given in addition to midazolam. In cases of continuing distress and where sedation is still inadequate, other drugs, such as ketamine, clonidine, fentanyl, lorazepam, propofol, and alimemazine are added. When pain is also suspected, as defined by a high numeric rating scale score (NRS ≥4), additional morphine is given. All study centers use this protocol, with only local differences in the choice of additional agents to midazolam and morphine (Additional file 1: Appendix).

#### Intervention group

**Figure 1:**
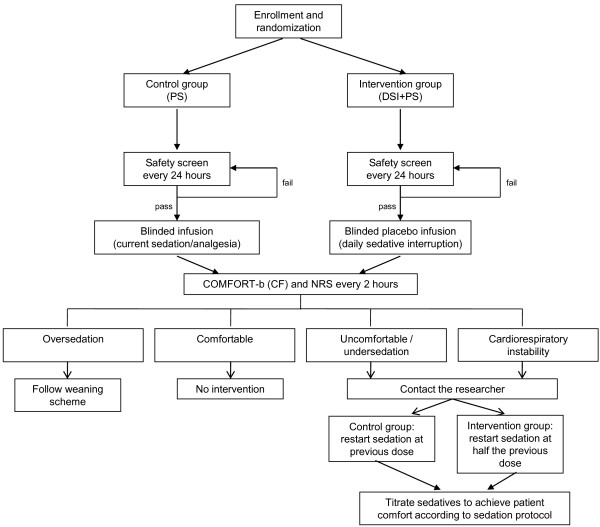
Flowchart of study design.

After the first 24 h of mechanical ventilation, the patient is assessed for a safety screen every morning at 10.00 AM, after routine care. This safety screen ensures that interruption of sedation is safe for the patient. If the patient passes the screen, the sedative/analgesic infusions will be discontinued immediately; this can be delayed for planned procedures. Analgesics needed for active pain will be continued (for example, pleural drain, <24 h after surgery). A patient passes the screen unless: he/she receives a sedative infusion for active seizures, receives escalating sedative doses due to ongoing agitation, receives neuromuscular blockers, has evidence of increased intracranial pressure or if there is cardiorespiratory instability. Patients who fail the test will be reassessed after 24 h (Figure 1).

During interruption, the patient may wake up, and therefore, patients will be monitored frequently. Patient comfort will be assessed routinely every 2 h using the COMFORT-b/NISS and NRS scores and at any time the patient appears distressed. The COMFORT-b score will be used to assess the level of sedation/wakefulness.

The sedative infusion will be started again: (a) if the patient becomes uncomfortable or agitated, according to the sedation protocol; or (b) if deemed necessary by the clinical team for instability in cardiorespiratory parameters, defined as the need to increase the ventilatory support or cardiovascular treatment (inotropes/fluid bolus), not associated with the underlying disorder.

After a loading dose of midazolam (0.1 mg/kg, intravenously), the sedative infusions will be restarted at half the previous dose and then titrated according to the sedation protocol by the nurse to achieve adequate sedation.

#### Control group

In the control patients, following the safety screen, a blinded infusion will be started at the same rate and dose as the patient was receiving. The level of sedation will be assessed in a manner similar to the interruption group. When assessments indicate distress, the study infusion will be ceased and replaced by the sedative infusion at a similar rate as before the interruption.

#### Blinding

Complete blinding after randomization was considered unsafe. It would mean blinded multiple infusion concentration/rate changes over time, leaving patients prone to drug dosing mistakes. However, during interruption, all patients will receive one or more blinded infusions (placebo in the intervention group and current sedation in the control group) prepared by the study pharmacist to minimize bias. In this way, the caregiving nurse will be blinded for placebo or current sedation during the interruption period. This will minimize bias in assessing the sedation level of the patient. At the end of the interruption period, the caregiving nurse will open the envelope that is placed in the study binder at the patient’s bedside to identify group allocation and sedation will be resumed at 50% (intervention group) or 100% (control group) of the previous intravenous infusion rate. This infusion rate is visible for the caregiving nurse and is therefore not blinded. This procedure will be repeated on every study day.

### Follow-up

Quality of life and symptoms of PTSD will be assessed 3 months after pediatric intensive care treatment using validated questionnaires.

Patients will be approached by telephone by the investigators. Quality of life will be determined using the Child Health Questionnaire (CHQ). The CHQ is a generic health profile measure covering physical and psychosocial domains that refer to the perceived health status for the collective 4 weeks prior to completing the questionnaire. Its structure and methodological approach are similar to those of the Short Form 36 (SF-36) scale, the most used quality of life measure in adults. We will ask parents to complete the CHQ for patients aged 2 months to 18 years. Patients aged 12 to 18 years will also be asked to complete the CHQ by themselves.

Symptoms of posttraumatic stress will be measured with the Dutch Children’s Responses to Trauma Inventory (CRTI). This is a 26-item self-report questionnaire for children aged 8 to 18 years. The questionnaire covers three subscales (intrusion, avoidance, hyperarousal) according to the diagnostic symptoms as per the *Diagnostic and Statistical Manual of Mental Disorders*, fourth edition (DSM-IV) for PTSD and one subscale for non-specific reactions. The total score of symptoms of PTSD can be used as an overall index of a child’s stress reaction following a stressful event.

### Endpoints

The main study endpoint is the number of ventilator-free days at 28 days, defined as the number of days a patient breathes without mechanical assistance for at least 48 h consecutively from day 1 to day 28 after randomization.

Secondary outcomes are: total and average dose of midazolam and morphine administered (mg/kg); number of COMFORT behavior scores <11 (oversedation) and >22 (undersedation); use of additional sedative or analgesic drugs during ventilation; total number of safety screen assessments and number and reason for failure to pass; total number and reasons for protocol deviations; adverse events (autoextubation and reintubation, accidental displacement of catheters and feeding tubes, pain, changes in blood pressure, heart rate, respiratory rate or alarms in those parameters (bradycardia/apneas) that need medication or adjustments in ventilator settings, need for soft wrist restrainers); incidence of withdrawal symptoms (Sophia Observation withdrawal Symptoms (SOS) scale); length of stay in the intensive care unit (days); length of stay in the hospital (days); organ failure free days, defined as the number of days from day 1 to day 28 in which the patient is without clinically significant organ dysfunction (the Paediatric Logistic Organ Dysfunction score (PELOD) will be used to define pediatric organ dysfunction); 30-day mortality; costs at 28 days; quality of life at 3 months, assessed by the Child Health Questionnaire; and incidence of PTSD at 3 months.

### Statistical methods

#### Sample size calculation

Our institutional admission data from 2008 showed that 168 children were mechanically ventilated for at least 48 h in our pediatric intensive care unit with mean ventilator-free days of 16.5 days (SD 9.9). Using these data, we calculated that a sample size of 100 patients per group is sufficient to detect a clinically significant difference of 25% in ventilator-free days (that is, mean 12.4 days in the intervention versus 16.5 days in the intervention group), with a power of 80%, based on a 2-tailed Mann–Whitney test with a significance level of 5%.

#### Final evaluation

Data will be analyzed with an intention-to-treat approach.

Demographic and clinical characteristics will be described using standard statistical analysis methods. Descriptive data will be presented as percentages, means ± SD for normally distributed variables, and medians ± interquartile ranges for non-normally distributed variables.

We will use *χ*^2^ tests or Fisher’s exact tests to compare the distribution of categorical variables between the study groups, and the Mann–Whitney test to compare continuous variables, including the primary outcome ventilator-free days.

The number of ventilator-free days will also be compared with correction for baseline variables (age, sex, PELOD score and type of disease), using multiple linear regression analysis.

To compare the effects of the two treatment protocols on length of stay in the intensive care unit and in the hospital, time-to-event analysis will be used. Kaplan-Meier analysis and the log-rank test will be used to assess the effect of the treatment protocols. These tests will also be used to assess the effect of the treatment on 30-day mortality. Cox proportional hazards analysis will be used to assess differences between the study groups after adjustment for the baseline variables mentioned previously.

All statistical tests will be two-tailed and the significance level will be set at 0.05.

### Ethical considerations

The study protocol has been evaluated and approved by the institutional review board of Erasmus Medical Centre, Rotterdam and by the local ethics committees of all participating centers: Radboud University Nijmegen Medical Centre, Academic Medical Centre of Amsterdam, Leiden University Medical Centre and University Medical Centre Groningen.

Written parental consent will be obtained from participants. The study will be conducted according to the principles of the declaration of Helsinki (version 2004) and in accordance with the Medical Research Involving Human Subjects Act (WMO).

This trial is registered in the Dutch Trialregister, located at http://www.trialregister.nl, under number NTR2030.

### Data and Safety Monitoring Board (DSMB)

All adverse events reported spontaneously by the subject or observed by the investigator or staff will be recorded. A continuous evaluation on adverse effects will be performed by an independent DSMB. Adverse events are defined as any undesirable experience occurring to a subject during the clinical trial. If it appears that a disproportionate number of adverse events occur in the intervention group, the DSMB can decide that the study must be terminated.

## Trial status

The trial is currently enrolling patients. We expect to finish patient recruitment in 2014.

## Abbreviations

DSI: daily sedation interruption; ICU: intensive care unit; PICU: pediatric intensive care unit; COMFORT-b: COMFORT behavior score; NISS: Nurse Interpretation of Sedation Score; NRS: numeric rating scale; ECMO: extracorporeal membrane oxygenation; PTSD: post-traumatic-stress disorder; CHQ: Child Health Questionnaire; DSMB: Data and Safety Monitoring Board.

## Competing interests

The authors declare that they have no competing interests.

## Authors’ contributions

NJV, SNdW, and MdH participated in the design and coordination of the study, the statistical plan and drafted this manuscript. CWMV and MGM participated in performing the study. CAJK was involved in drafting the concept of the study. WCJH and JvR advised and performed power calculation and statistical plan development. DT supervised all aspects of the study. All authors have read and approved the final manuscript.

## Supplementary Material

Additional file 1 **Appendix.** Sedation protocol. Click here for file

## On behalf of SKIC study group

Nijmegen: Joris Lemson, Amsterdam: Job B van Woensel, Leiden: PP Roeleveld, Utrecht: Nicolaas JG Jansen, Groningen: Martin C Kneyber.
