# Supplementary material for: Daily interruption of sedation in critically ill children: study protocol for a randomized controlled trial
Source: Trials. 2014 Feb 13;15:55. doi: 10.1186/1745-6215-15-55 (PMC3928607; doi:10.1186/1745-6215-15-55)

Appendix 1. Sedation protocol, basic scheme

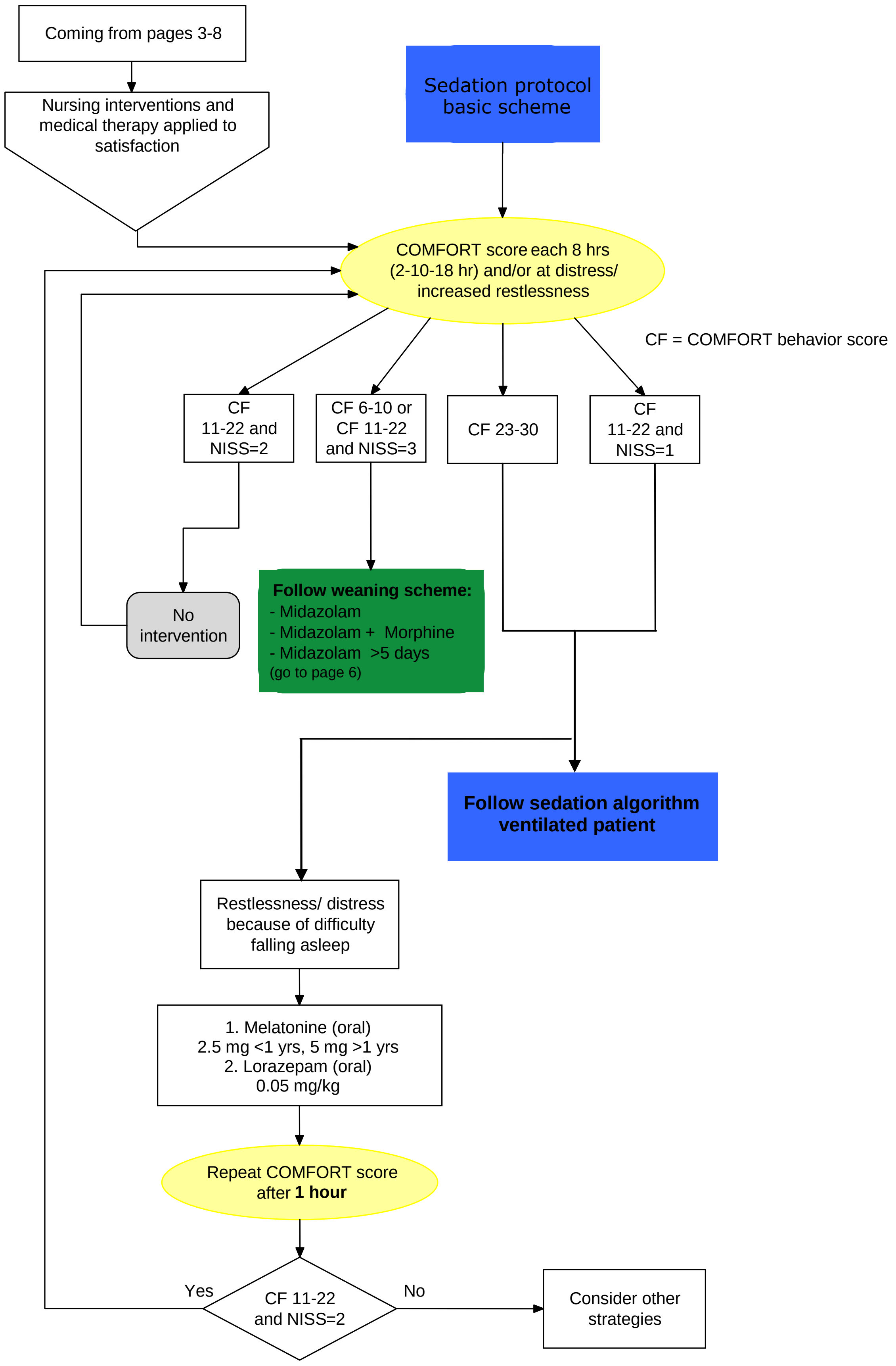

Appendix 2. Sedation protocol, increasing decision tree

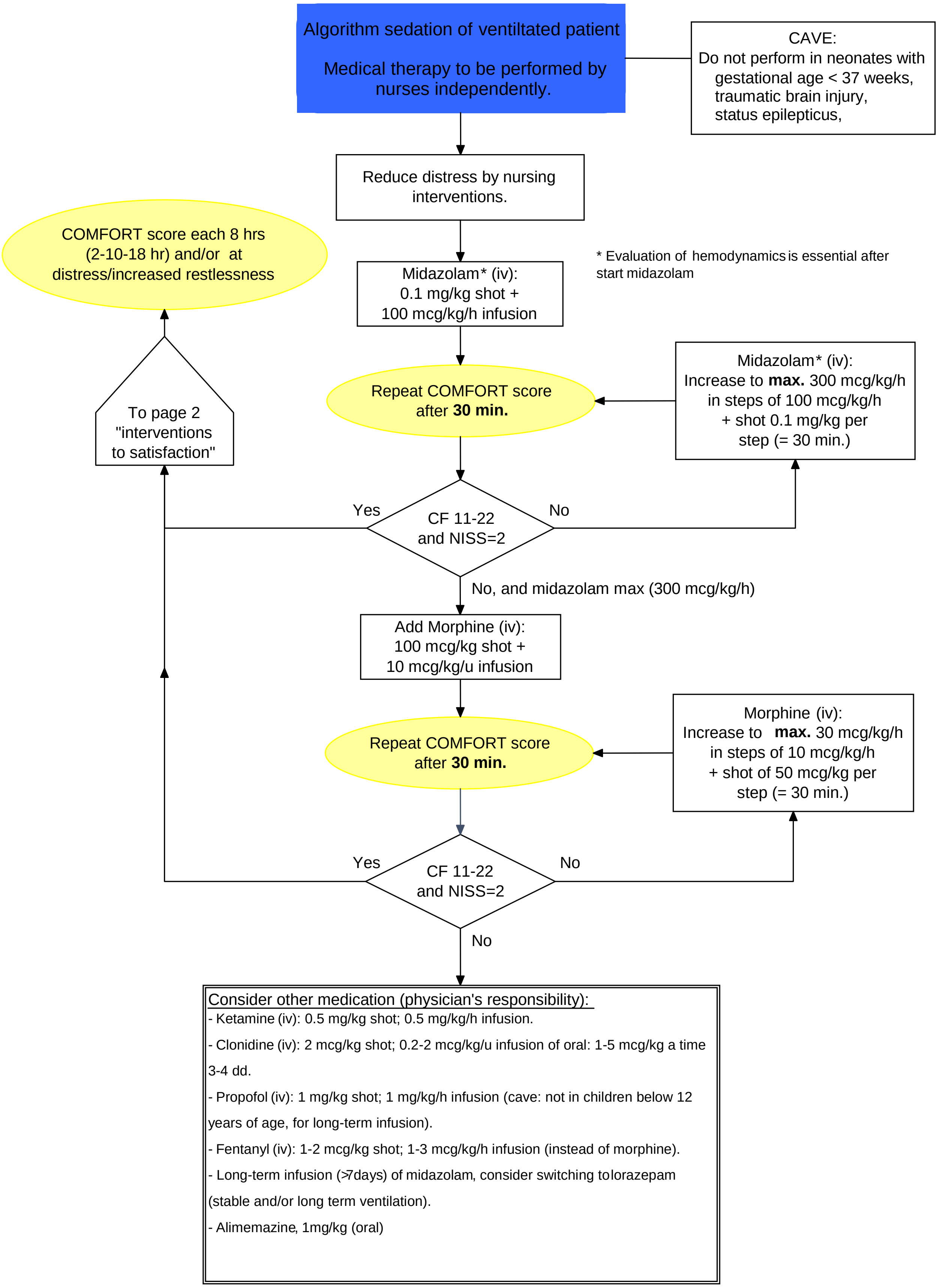

Supplement: Additional file 1 — Appendix. Sedation protocol. [file 1745-6215-15-55-S1.pdf]
